# Hippocampus-sparing volume-modulated arc therapy in patients with World Health Organization grade II glioma: a feasibility study

**DOI:** 10.3389/fonc.2024.1445558

**Published:** 2025-01-20

**Authors:** Renxian Xie, Hongxin Huang, Qingxin Cai, Jiayang Lu, Tong Chen, Keyan Xie, Jianzhou Chen, Chuangzhen Chen

**Affiliations:** ^1^ Department of Radiation Oncology, Cancer Hospital of Shantou University Medical College, Shantou, China; ^2^ Shantou University Medical College, Shantou, China

**Keywords:** hippocampus-sparing, volumetric-modulated arc therapy, glioma, dosimetric feasibility, World Health Organization grade II

## Abstract

**Background:**

Radiotherapy can improve the survival rates of patients with glioma; meanwhile, impaired cognitive functions have been brought to the forefront with the offending organ, the radiosensitive hippocampus. This study aimed to assess the feasibility of hippocampus-sparing volumetric-modulated arc therapy (HS VMAT) in patients with World Health Organization (WHO) grade II glioma.

**Methods:**

HS VMAT plans and non-hippocampus-sparing volumetric-modulated arc therapy (NHS VMAT) plans were generated using a computed tomography (CT) dataset of 10 patients who underwent postoperative radiotherapy. The dose volume histogram (DVH), homogeneity index (HI), conformity index (CI), and irradiated dose of the hippocampus and other organs at risk (OARs) were analyzed.

**Results:**

No significant differences were observed in HI and CI between the two plans. Regarding the protection of OARs, HS VMAT plans were equally capable and even lowered the radiation dosages to the brainstem (35.56 vs. 41.74 Gy, p = 0.017) and spinal cord (1.34 vs. 1.43 Gy, p = 0.006). Notably, HS VMAT plans markedly decreased doses to the ipsilateral hippocampus and the contralateral hippocampus, demonstrating its efficacy in hippocampal dose reduction.

**Conclusion:**

The HS VMAT plan can be used to efficiently lower the dosage delivered to the hippocampus and may, to some extent, help lessen the risk of cognitive damage. The encouraging results of our study need to be further validated by clinical trials to confirm the benefits of the HS VMAT plans in preserving cognitive functions in patients with glioma.

## 1 Introduction

At present, gliomas, akin to those of regular glial cells histologically, are still one of the most common original malignant brain tumors (1). According to the Central Brain Tumor Registry of the United States (CBTRUS), there were 106,808 patients diagnosed with glioma in the USA from 2015 to 2019 (2). The World Health Organization (WHO) has classified glioma into four grades based on their pathological appearances, and the concept of low-grade glioma was defined as grade I or II glioma (3, 4). Low-grade gliomas are customarily deemed to be benign, for the symptoms are rare apart from seizures, but there is potential for cancerous growth yet (5, 6). The comprehensive treatment for low-grade glioma consists of surgery, chemotherapy, radiotherapy, and immunotherapy (7). Among these, radiotherapy (RT) is one of the clinically routine therapies for glioma, including conformal RT, intensity-modulated RT, stereotactic radiosurgery (SRS), et al. (8). Several studies have confirmed that RT can improve the survival rates of patients with glioma (9, 10). However, there are also side effects caused by RT, among which impaired cognitive functions have been brought to the forefront. Long-term follow-up research showed that receiving RT could result in impairments in attentional and cognitive functions in patients with low-grade glioma (11). High radiation dosages could result in defects in language fluency, executive capability, and speed in processing information (12). Moreover, an intervention review conducted by Theresa et al. drew a similar conclusion that patients with glioma who undergo RT may be more likely to experience cognitive impairments (13). In a prospective study that evaluated the hippocampal radiation dosages with cognitive functions’ changes after whole brain radiotherapy (WBRT) (14), the hippocampus was regarded as an essential construction for normal cognition in humans, which could be easily injured during RT and takes responsibility for cognitive impairments in these patients. Due to a more favorable prognosis and extended survival compared with patients with high-grade glioma, individuals diagnosed with low-grade glioma face an elevated risk of cognitive impairments. Reducing the radiation dose to the hippocampus helps protect patients’ cognitive functions (12), but the hippocampus is not usually considered an organ at risk (OAR) in clinical practice. A retrospective review conducted by Pinkham MB et al. revealed that hippocampal-sparing intensity-modulated radiotherapy (IMRT) can limit hippocampal Dmean to less than 30 Gy on at least one side of the hippocampus while maintaining the doses necessary for treating WHO grade II and III glioma (15).

In this study, hippocampal-sparing was defined as maintaining an average dose to the ipsilateral hippocampus adjacent to the tumor at no more than 30 Gy. Concurrently, the dose administered to the entirety of the contralateral hippocampus was restricted to not exceed 9 Gy, with the maximum dose constrained to remain below 16 Gy. Several techniques have been investigated in hippocampus-sparing VMAT for whole brain irradiation. Head-tilting technique adjusts the tilt angle of head by using a baseplate, leading to the removal of the lens from the beam pathway (16, 17). This method has demonstrated efficacy in hippocampal protection by significantly lowering the radiation dose to the hippocampus, which in turn reduces the potential for cognitive impairment. Split-arc partial-field VMAT (sapf-VMAT) techniques can also further reduce the dose to the hippocampus (18). The expansive irradiation field required for the whole brain target volume requires a broader jaw opening. This sapf-VMAT technique reduces the field size and solves the island blocking problem, which exists when ≥ 2 areas of whole brain target volume share the same multi-leaf collimator (MLC) leaf pair, and therefore it can shield the hippocampus more effectively. However, for gliomas, the target area is relatively small, the irradiation field only requires relatively small jaw opening. Hybrid sapf-VMAT with an improved beam arrangement ensures adequate dose delivery to the tumor target area while minimizing exposure to the hippocampus and other critical organs, providing a more beneficial treatment approach for patients (19).

The IMRT technique is routinely used in the treatment of glioma. However, a growing number of studies have found that volume-modulated arc therapy (VMAT) further improves dose conformity, reduces the radiation dose to surrounding normal tissues, and shortens treatment time compared with IMRT (20, 21). Beyond our expectation, there is no further research exploring whether we can minimize the radiation dose to the hippocampus of patients with WHO grade II glioma for the purpose of preserving their cognitive functions while using VMAT as the treatment.

Regarding the above-mentioned issue, this study aimed to assess the feasibility of hippocampus-sparing volumetric modulated arc therapy (HS VMAT) in patients diagnosed with WHO grade II glioma pathologically.

## 2 Materials and methods

### 2.1 Patient characteristics

The CT datasets of ten patients with WHO grade II glioma diagnosed at the Cancer Hospital of Shantou University Medical College between 2014 and 2023 were included in this dosimetric planning analysis. **Table 1** lists the characteristics of these cases, including gender, histological types of glioma, location, clinical target volume (CTV), ipsilateral hippocampus volume, and contralateral hippocampus volume. In all cases, the tumors were localized in either the left or right hemisphere. Therefore, a clear region of the ipsilateral and contralateral hippocampus could be delineated.

**Table 1:** Characteristics of 10 patients with WHO grade II gliomas.

Case	Gender	Histology	Location	CTV (mm^3^)	IH Volume (mm^3^)	CH Volume (mm^3^)
1	M	Adult-type astrocytoma	Left frontal lobe	192.7	1.4	1.6
2	M	Oligodendroglioma	Frontal lobe	201.0	2.3	2.1
3	M	Diffuse astrocytoma	Left parietal lobe	183.4	3.0	2.8
4	F	Neuroastrocytoma	Left thalamus	119.3	1.6	2.9
5	F	Oligodendroglioma	Right frontal lobe	203.7	2.9	2.8
6	F	Oligodendroglioma	Right frontal lobe	187.7	3.7	3.8
7	F	Diffuse astrocytoma	Left temporo-parietal lobe	205.8	2.9	2.8
8	F	Gemistocytic astrocytoma	Right temporal lobe	171.1	2.1	2.0
9	F	Diffuse astrocytoma	Left temporo-parieto-occipital lobe	283.7	3.0	3.1
10	F	Neuroastrocytoma	Left frontal-temporal lobe	322.2	2.3	2.3
Mean ± SD				207.1 ± 57.1	2.5 ± 0.7	2.6 ± 0.6

CTV, Clinical Target Volume; IH, ipsilateral hippocampus; CH, Contralateral Hippocampus; M, Male; F, Female; SD, Standard Deviation.

### 2.2 CT simulation

All patients were positioned in the supine position, while their heads were immobilized using a specialized thermoplastic cast. CT scans (with a slice thickness of 3 mm) were conducted from the uppermost region of the cranium’s scalp to the first cervical vertebrae (Philips Brilliance CT Big Bore Oncology Configuration, Cleveland, OH) using intravenous contrast injection. The aforementioned CT images were subsequently transferred to the Eclipse version 15.6 treatment planning system (Varian Medical System, Inc., Palo Alto, CA, USA) to enable target and organ-at-risk delineation as well as treatment planning.

### 2.3 Target delineation and OAR definition

All target volumes and organs at risk (OARs) were delineated by our radiation oncologists according to the 2022 NCCN Guidelines^®^ in low-grade glioma. Targets and the OARs were localized on the basis of the CT images and modified according to the pre-treatment MRI images in fusion. Tumor volumes are defined using pre- and postoperative MRI imaging, usually T2 fluid-attenuated inversion recovery (FLAIR) and T1 post-contrast sequences, to define gross tumor volume (GTV). The CTV encompassed the surgical cavity, any residual regions exhibiting gadolinium enhancement as observed on T1-weighted images, and areas of hyperintensity identified on T2-weighted images, with an additional 1.5 cm margin to account for potential microscopic invasion. The CTV was delineated to exclude natural barriers, such as bone and the falx cerebri. The planning target volume (PTV) was defined by expanding the CTV by 0.3 cm in all directions. The hippocampal structure was delineated in accordance with the RTOG 0933 hippocampal atlas (22). The hippocampal structure was delineated in accordance with the RTOG 0933 hippocampal atlas. The hippocampal avoidance region was created by extending the hippocampal contour with a volumetric margin of 5 mm. In this study, the OARs were contoured following the 2022 NCCN Guidelines^®^, encompassing the eyeballs, lenses, optic nerves, optic chiasm, pituitary gland, and spinal cord. Notably, the hippocampus was classified as an OAR exclusively in the HS VMAT plans. When the PTV overlaps with critical structures like the brainstem and spinal cord, dose limitation to these OARs is prioritized. If the overlap involves less critical OARs like lenses, eyeballs, and the hippocampus, ensuring adequate PTV coverage takes precedence.

### 2.4 VMAT planning

The VMAT technique using 6-MV photons from a TrueBeam (Varian Medical System, Inc., Pao Alto, CA) linear accelerator was employed on these patients. The VMAT plans using coplanar dual half arcs or dual full arcs with collimator 330°/30° were generated in Eclipse version 15.6 (Varian Medical Systems, Palo Alto, CA, USA) treatment planning systems. The median value of field size in X direction was 11.05 (range from 9.1 to 14.9) cm. Dose-limiting ring structures were generated to form the dose gradients surrounding the PTV. The Photon Optimizer (PO, version 15.6.06) algorithm was used for plan optimization. To avoid bias, the optimization objectives for both techniques were kept the same for each patient except that the priorities of the hippocampus were set as 0 for non-hippocampus-sparing (NHS) VMAT. Details of which can be found in the **Supplementary Material**. The Anisotropic Analytical Algorithm (AAA, version 15.6.06) was applied for final dose calculations, with a grid size of 2.5 mm. Each plan was normalized such that the prescribed dose covered 95% of PTV.

In the VMAT plans in this study, the prescribed dose for these patients was a total of 54 Gy administered in 30 fractions.

### 2.5 Plan evaluation

The aim of the treatment was to ensure that the PTV received a minimum of 95% and a maximum of 110% of the prescribed dose while minimizing the radiation exposure to the rest of the brain. The attainment of HS VMAT plans was determined by ensuring that the mean dose to the ipsilateral hippocampus did not exceed 30 Gy (23). Simultaneously, the dosage constraints for the contralateral hippocampus within this investigation mirrored those delineated in the RTOG 0933 study (22). Specifically, the dosage to 100% of the contralateral hippocampus was not permitted to surpass 9 Gy, and the maximum hippocampal dosage was capped at 16 Gy. Any dosage to 100% of the contralateral hippocampus that exceeded 10 Gy, or a maximum hippocampal dosage surpassing 17 Gy, was deemed an intolerable deviation, necessitating the revision of the treatment plan. Adequate coverage of the PTV was never compromised to achieve sufficient sparing of the hippocampal structures. Dose constraints to other OARs (such as the optic chiasm and brainstem) were prescribed as clinically indicated and prioritized over hippocampal constraints.

The evaluation of the treatment plans was conducted by considering the following parameters: dose volume histogram (DVH), homogeneity index (HI), conformity index (CI), PTVminus volume (defined as the planning target volume excluding the overlap with the brain stem PRV), PTVminus D98 (the dose covering 98% of the PTVminus volume), PTVminus D50 (the dose covering 50% of the PTVminus volume), PTVminus D2 (the dose covering 2% of the PTVminus volume), and PTVminus V54 (the volume covered by the prescription dose, excluding the overlap with the brain stem PRV). Given the brain stem’s vital role, we added a 0.3-0.5 cm buffer around it, termed the brain stem PRV.

The following formulas were used for the calculation of the HI and the CI:


(1) $$HI=\frac{D2-D98}{D50}$$

(2) $$CI=\frac{TV_{RI}}{TV}\times \frac{TV_{RI}}{V_{RI}}$$

In this study, D2, D50, and D98 denote the doses encompassing 2%, 50%, and 98% of the target volume, respectively. The prescribed dose across all treatment plans is 54 Gy. For the PTV, D2 and D98 were selected as representative of the approximate maximal and minimal doses, respectively, to assess regions of higher and lower dose concentrations, commonly referred to as hot and cold spots. The target volume (TV) covered by the reference isodose is denoted as TVRI, while VRI represents the volume of the reference isodose (24). Ideally, the homogeneity index (HI) should approach 0, signifying improved dose homogeneity with lower values, whereas the conformity index (CI) should ideally be 1, indicating enhanced conformality with higher values.

The parameters utilized to assess the radiation dosage in the ipsilateral, contralateral, and bilateral hippocampus encompassed Dmax, Dmean, Dmin, D100, and D40. The planning constraints for the OARs are specified as follows: Dmax should be less than 60 Gy for the brainstem PRV, less than 54 Gy for the brainstem, less than 8 Gy for the lens, and less than 45 Gy for the spinal cord; Dmean should be less than 50 Gy for the pituitary gland, and Dmax should be less than 50 Gy for the eyeballs, optic nerves, and optic chiasm.

In this context, Dx denotes the dose level that is achieved or surpassed in x% the specified volume. Meanwhile, V100% refers to the volume that receives a minimum of 100% of the prescribed dose. Additionally, Dmax signifies the maximum dose administered, whereas Dmean indicates the average dose delivered.

## 3 Results

### 3.1 Target coverage, conformity and dose homogeneity

**Figure 1:**
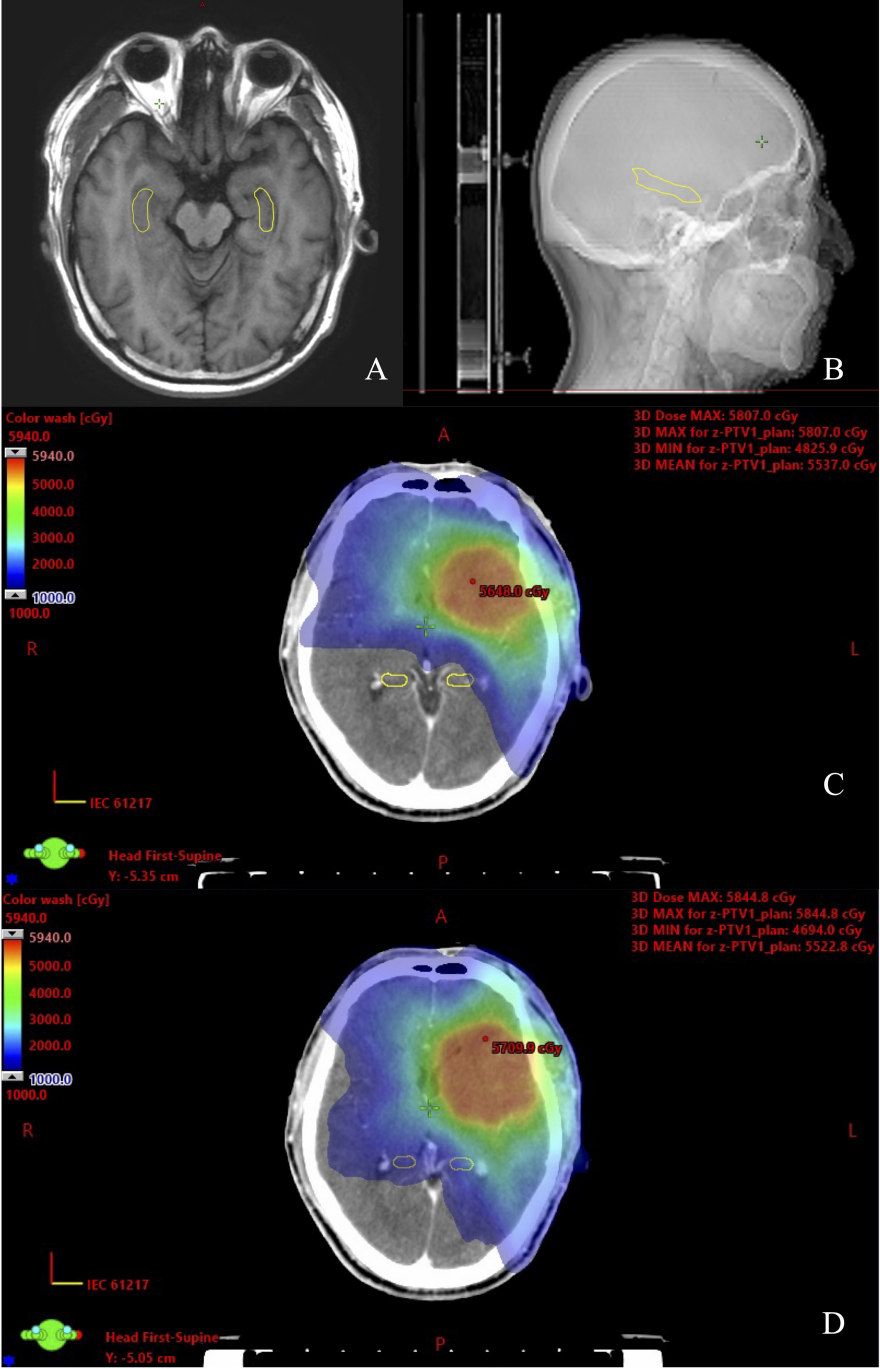
View of dose distribution of the hippocampus contoured on CT-MRI fusion for one glioma case. **(A)** Hippocampal contour in transverse plane. **(B)** Hippocampal contour in sagittal plane. **(C)** HS VMAT plans. **(D)** NHS VMAT plans.

**Figure 2:**
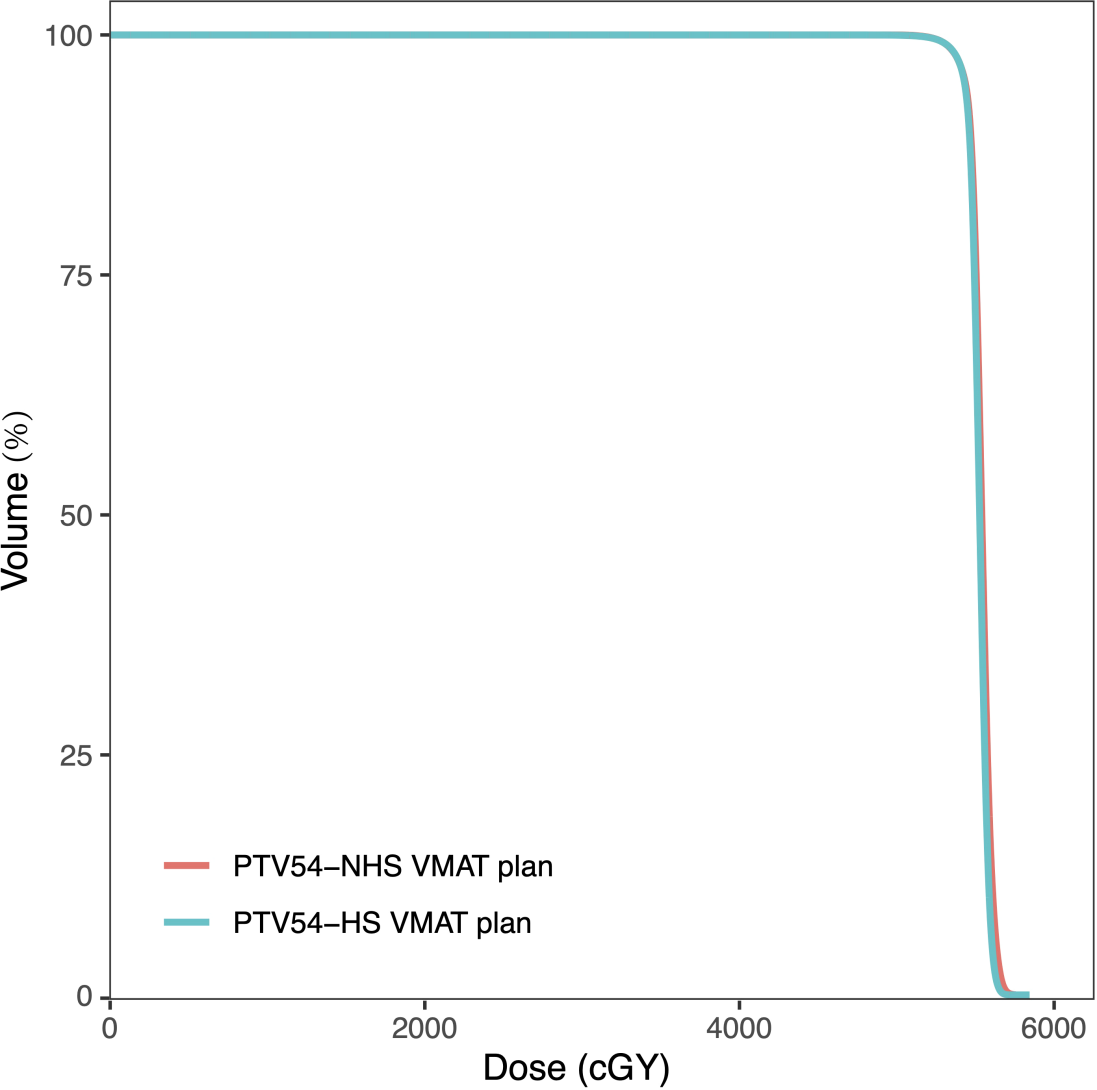
Dose-volume histograms (DVHs) comparison of PTV54 in one glioma case for NHS VMAT plans and HS VMAT plans.

All of the HS VMAT plans and NHS VMAT plans were clinically acceptable. The dose distributions for the NHS VMAT plans and HS VMAT plans in one patient are shown in **Figure 1**. **Table 2** displays the simulated radiation dose for the PTVminus in both plans. The CI and HI analyses were analyzed utilizing the paired-sample t-test. The HI (0.057 ± 0.014 vs. 0.046 ± 0.009, p=0.011*) and CI (0.91 ± 0.02 vs. 0.92 ± 0.02, p=0.071) of HS VMAT plans had no significant differences compared with NHS VMAT plans. The DVH of the PTV54 in one glioma case for NHS VMAT plans and HS VMAT plans is shown in **Figure 2**. The above results indicated that the HS VMAT plans were not inferior to the traditional NHS VMAT plans in terms of dose distribution.

**Table 2:** Dosage distribution in PTVminus in NHS VMAT plans and HS VMAT plans.

	NHS VMAT (Mean ± SD)	HS VMAT (Mean ± SD)	*P*-value
PTVminus			
D98 (Gy)	53.51 ± 0.16	53.30 ± 0.35	0.016^*^
D50 (Gy)	55.05 ± 0.28	55.27 ± 0.27	0.022^*^
D2 (Gy)	56.03 ± 0.50	56.48 ± 0.51	0.018^*^
HI	0.046 ± 0.009	0.057 ± 0.014	0.011^*^
CI	0.92 ± 0.02	0.91 ± 0.02	0.071

NHS VMAT, non-hippocampus-sparing volume modulated arc therapy; HS VMAT, hippocampus-sparing volume modulated arc therapy; PTVminus, PTV minus the overlapping with Brain stem PRV; D98, the dose covered 98% of target volume; D50, the dose covered 50% of target volume; D2, the dose covered 2% of target volume; HI, homogeneity index; CI, conformity index; *: P<0.05, SD, Standard Deviation.

### 3.2 OARs

**Figure 3:**
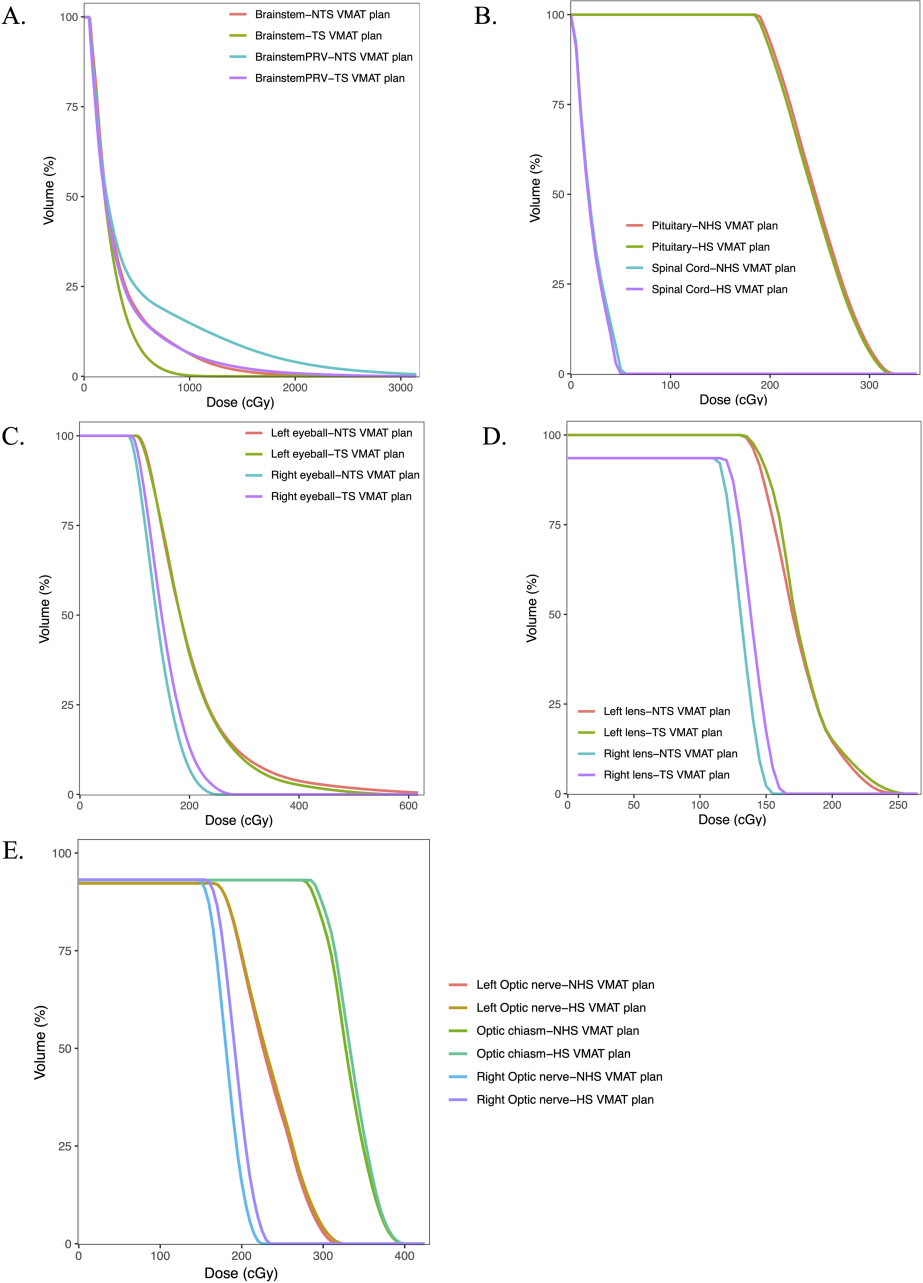
Dose-volume histograms (DVHs) comparison of OARs in one glioma case for NHS VMAT plans and HS VMAT plans: **(A)** The dose-volume histogram for brainstem and brainstem PRV, **(B)** The dose-volume histogram for pituitary and spinal cord, **(C)** The dose-volume histograms for eyeballs, **(D)** The dose-volume histogram for lens, **(E)** The dose-volume histogram for optic nerves and optic chiasm.

The dosimetric outcomes for the various OARs in each treatment plan are presented in **Table 3**. In comparison to the standard VMAT plans, the HS VMAT plans demonstrated a statistically significant reduction in radiation doses to the brainstem (35.56 ± 16.67 vs. 41.74 ± 16.18, p=0.017) and spinal cord (1.34 ± 0.77 vs. 1.43 ± 0.80, p=0.006), without a significant increase in radiation exposure to other OARs, including the pituitary gland, eyeball, lens, and optic nerve. Despite notable differences between the two plans concerning the OARs for the right eyeball, left lens, and right optic nerve, the dosage constraints for these OARs complied with established guidelines. **Figure 3** illustrates the DVH of the OARs in a glioma case for both NHS VMAT and HS VMAT plans.

**Table 3:** Dosage distribution in OARs in NHS VMAT plans and HS VMAT plans.

	NHS VMAT (Mean ± SD)	HS VMAT (Mean ± SD)	*P*-value
Brain stem PRV			
Dmax (Gy)	48.16 ± 12.94	44.41 ± 15.52	0.285
Brain stem			
Dmax (Gy)	41.74 ± 16.18	35.56 ± 16.67	0.017^*^
Left eyeball			
Dmax (Gy)	19.72 ± 11.43	19.56 ± 11.54	0.333
Right eyeball			
Dmax (Gy)	19.81 ± 16.18	21.64 ± 16.35	0.047^*^
Left lens			
Dmax (Gy)	4.80 ± 1.42	5.22 ± 1.67	0.013^*^
Right lens			
Dmax (Gy)	4.65 ± 1.41	4.75 ± 1.44	0.386
Left optic nerve			
Dmax (Gy)	23.11 ± 19.13	23.75 ± 19.75	0.139
Right optic nerve			
Dmax (Gy)	15.89 ± 12.12	17.72 ± 12.28	0.037^*^
Optic chiasm			
Dmax (Gy)	31.90 ± 19.05	30.68 ± 19.04	0.508
Pituitary			
Dmax (Gy)	20.46 ± 14.53	18.26 ± 12.05	0.307
Spinal cord			
Dmax (Gy)	1.43 ± 0.80	1.34 ± 0.77	0.006^*^

NHS VMAT, non-hippocampus-sparing volume modulated arc therapy; HS VMAT, hippocampus-sparing volume modulated arc therapy; PRV, planning organ at risk volume; Dmax, maximum dose; *: P<0.05, SD, Standard Deviation.

### 3.3 Ipsilateral, contralateral and bilateral hippocampus

**Figure 4:**
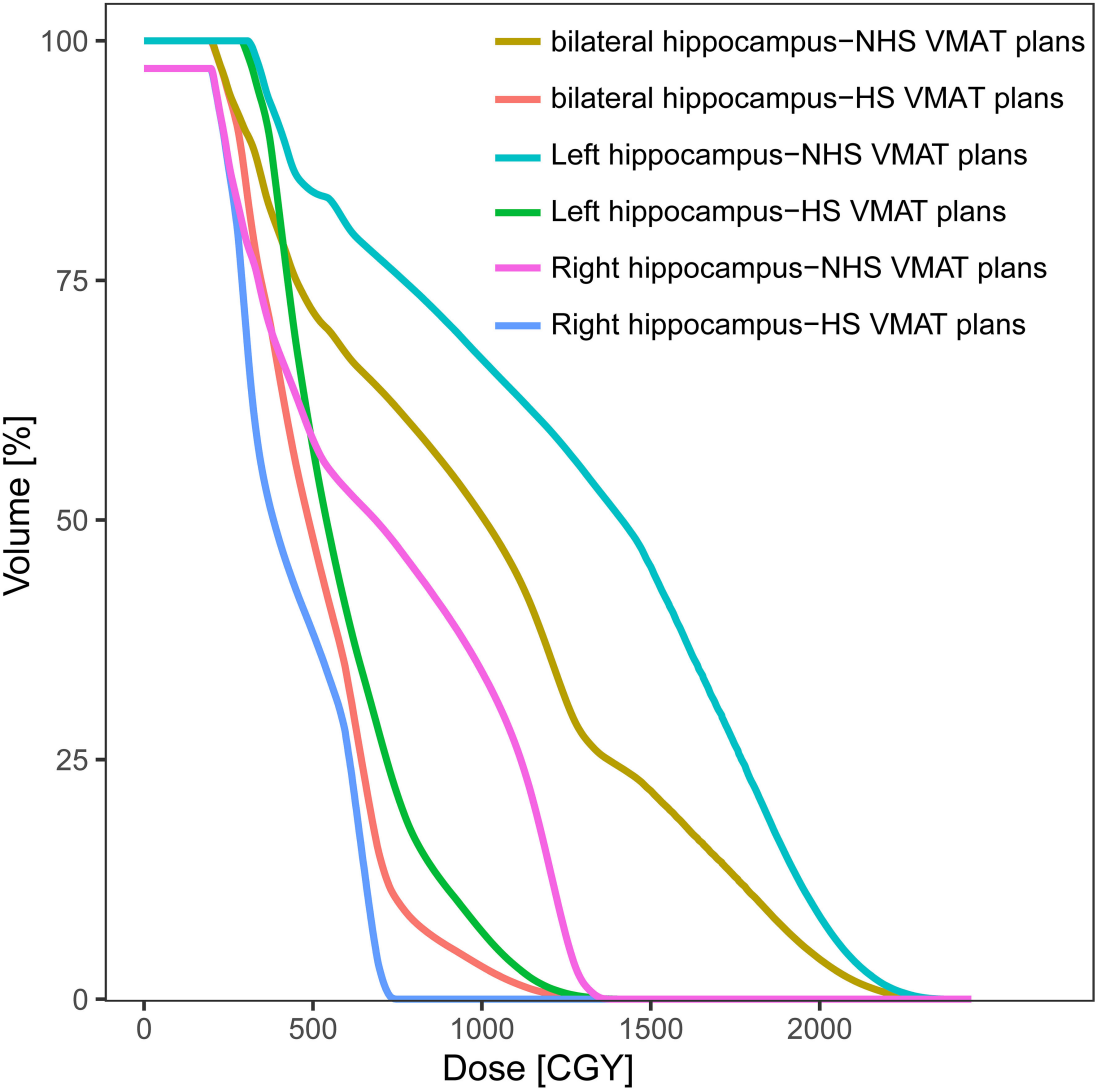
Dose-volume histograms (DVHs) comparison of hippocampus in one glioma case for NHS VMAT plans and HS VMAT plans.

The radiation dosages of the hippocampus were much lower in HS VMAT plans when compared with NHS VMAT plans. The accumulated dose to the ipsilateral, contralateral, and bilateral hippocampus under two different VMAT plans was analyzed. Details were reported in **Table 4**. For the ipsilateral hippocampus, HS VMAT plans reduced radiation doses to 7.80 ± 4.20 Gy (Dmin), 18.60 ± 8.52 Gy (Dmean), 34.28 ± 12.79 Gy (Dmax), 7.89 ± 4.08 Gy (D100), and 19.98 ± 9.63 Gy (D40) from higher doses seen in NHS VMAT plans. For the contralateral hippocampus, HS VMAT plans achieved reductions to 3.38 ± 1.12 Gy (Dmin), 4.86 ± 0.91 Gy (Dmean), 8.41 ± 1.38 Gy (Dmax), 3.42 ± 1.05 Gy (D100), and 4.89 ± 0.94 Gy (D40). Moreover, for the bilateral hippocampus, HS VMAT plans decreased radiation doses to 3.38 ± 1.12 Gy (Dmin), 11.52 ± 4.74 Gy (Dmean), 34.28 ± 12.79 Gy (Dmax), 3.42 ± 1.05 Gy (D100), and 12.54 ± 6.40 Gy (D40) compared to the higher doses in NHS VMAT plans. The DVH of the hippocampus in one glioma case for NHS VMAT plans and HS VMAT plans is shown in **Figure 4**.

**Table 4:** Dosage distribution in the hippocampus in NHS VMAT plans and HS VMAT plans.

	NHS VMAT (Mean ± SD)	HS VMAT (Mean ± SD)	*P*-value
Ipsilateral hippocampus			
Dmin (Gy)	22.33 ± 19.12	7.80 ± 4.20	0.005^*^
Dmean (Gy)	35.20 ± 19.58	18.60 ± 8.52	0.005^*^
Dmax (Gy)	45.37 ± 16.84	34.28 ± 12.79	0.013^*^
D100 (Gy)	22.33 ± 19.12	7.89 ± 4.08	0.005*
D40 (Gy)	36.73 ± 19.52	19.98 ± 9.63	0.005^*^
Contralateral hippocampus			
Dmin (Gy)	9.68 ± 6.51	3.38 ± 1.12	0.005^*^
Dmean (Gy)	16.86 ± 8.86	4.86 ± 0.91	0.006^*^
Dmax (Gy)	29.13 ± 13.88	8.41 ± 1.38	0.001^*^
D100 (Gy)	9.68 ± 6.51	3.42 ± 1.05	0.006*
D40 (Gy)	17.30 ± 9.14	4.89 ± 0.94	0.005^*^
Bilateral hippocampus			
Dmin (Gy)	9.68 ± 6.51	3.38 ± 1.12	0.005*
Dmean (Gy)	24.75 ± 14.90	11.52 ± 4.74	0.004*
Dmax (Gy)	45.37 ± 16.84	34.28 ± 12.79	0.013*
D100 (Gy)	9.68 ± 6.51	3.42 ± 1.05	0.006*
D40 (Gy)	31.98 ± 21.83	12.54 ± 6.40	0.005*

NHS VMAT, non-hippocampus-sparing volume modulated arc therapy; HS VMAT, hippocampus-sparing volume modulated arc therapy; Dmin, minimum dose; Dmean, mean dose; Dmax, maximum dose; D100, the dose covered 100% of target volume; D40, the dose covered 40% of target volume; *: P<0.05, SD, Standard Deviation.

## 4 Discussion

While radiotherapy has been established as a standard treatment for glioma, and patients with low-grade glioma have experienced improved long-term survival outcomes, it is imperative to minimize adverse effects, such as cognitive decline. This approach aims to enhance the quality of life for these patients.

This study focused exclusively on patients diagnosed with WHO grade II glioma, given that these lower-grade gliomas exhibit less aggressive and malignant characteristics compared to higher-grade gliomas. In contrast, patients with WHO grade III or IV glioma generally experience a limited survival period of only a few years post whole-brain radiotherapy (25–27). This duration may be inadequate for the emergence of significant cognitive deficits attributable to hippocampal damage, as such cognitive impairments typically develop over an extended period (28). In contrast, patients diagnosed with WHO grade II glioma may survive for several decades, during which hippocampal damage can result in considerable cognitive deterioration, imposing a substantial burden on both families and society. Since individuals with WHO grade I glioma typically do not require postoperative radiotherapy, they were excluded from this study (29).

The definite mechanisms of the decline in cognitive functions after radiotherapy remain unclear. Currently, there is a prevailing belief that radiation-induced damage to hippocampal neuron stem cells and alterations in the microenvironment may hold a significant influence. The dentate gyrus and subgranular zone of the hippocampi are the major regions where human neuron stem cells are, except for the subventricular zone (30). Physically, neuron stem cells undergo differentiation into granule cells in neurogenesis, resulting in the migration of these cells from the subgranular zone to the granular layer. These newly formed cells exhibit greater excitability compared to mature cells, enabling them to contribute to message integration, learning, memory, mood regulation, and other complex cognitive functions in humans (31). While undergoing RT, progenitor cells with greater potential for division and differentiation are more susceptible to influence, resulting in cognitive function impairments (30). Meanwhile, RT can induce the destabilization of the vascular endothelium, causing vascular rarefaction. Because of hippocampi’s dependence on blood flow integrity, decreased vascular density leads to incomplete differentiation of neuron stem cells, which could be reversed by systemic hypoxia (32). There are plenty of inflammatory cytokines accumulated in the hippocampi microenvironment after RT, including TNF-α, IL-1β, IL-6, NF-κb, prostaglandin E2, and so on, causing vascular injury and suppressing neurogenesis (33). In addition, inflammatory responses induced by RT may play irreplaceable roles in cognitive dysfunction. A novel finding revealed that radiation-induced NACHT, LRR, and PYD domain-containing protein 3 inflammasomes generate IL-1β, IL-18, and caspase-3 downstream, therefore causing hippocampal neuron pyroptosis. The above inflammatory signaling pathways could be inhibited by alternate-day fasting (34).

In this dosimetric feasibility study, ten patients with WHO grade II glioma were included. All of them had already been treated with standardized VMAT without hippocampus sparing. Therefore, bilateral hippocampi were defined as OARs, and we chose to generate inverse-planning VMAT with Eclipse version 15.6 retrospectively, owing to the restrictive dosage constraints in OARs. Evaluation of target dosage distribution in two radiotherapy plan indicators showed that there were no significant differences in HI and CI between the HS VMAT and the NHS VMAT plans. What excited us the most was the significant reduction in radiation dosages in the hippocampi area with the HS VMAT plans, including Dmax, Dmean, Dmin, D100, and D40. Gondi et al.’s research found that irradiating over 40% of the bilateral hippocampus with doses above 7.3 Gy led to long-term memory recall issues (35). However, in our study, it was not feasible to restrict the irradiation dose to less than 7.3 Gy for 40% of the bilateral hippocampus in all patients, due to the proximity of some tumors to the hippocampus and the presence of large tumor foci. Nonetheless, the HS VMAT plans effectively reduced the bilateral hippocampal D40 compared to the NHS VMAT plans. Furthermore, the HS VMAT plans significantly decreased radiation exposure to the brainstem and spinal cord, without substantially increasing the radiation dose to other OARs, such as the pituitary gland, eyeball, lens, and optic nerve, in comparison to the NHS VMAT plans. As for the brain stem, Dmax was 35.56 ± 16.67 Gy when using the HS VMAT plan, in contrast to 41.74 ± 16.18 in the NHS plan, with a P-value equal to 0.017. Additionally, Dmax in the spinal cord could be decreased to 1.35 ± 0.77 Gy in the HS VMAT plan, with a P-value of 0.006. This study revealed that the HS VMAT plan exhibited certain advantages over the NHS VMAT plan in radiotherapy for WHO grade II glioma.

The negative effects of irradiation on the hippocampi, or irradiation-tolerance, can be influenced by patient age, tumor location, radiation dose per fraction, interval between fractions, and specific radiotherapy techniques. When gliomas are near the hippocampi, the PTV may encroach on the protected area, potentially reducing the effectiveness of HS VMAT plans. It’s disappointing that there’s no agreed-upon dose limit for hippocampal radiation. A study by Tsai et al. found that administering over 7.45 Gy and 5.83 Gy to 50-100% of both hippocampi significantly affected verbal memory four months post-WBRT.[11]. According to a previous phase II study conducted by Gondi V et al. (22), the maximum dose to the hippocampus should be restricted to less than 16 Gy and the D100 to less than 9 Gy in patients with metastatic brain tumors receiving WBRT. However, the prescribed dose for a glioma radiotherapy plan is 54 Gy. Contemporary radiation treatments, including IMRT and VMAT, have considerable hurdles in reducing the maximum dosage to the tumor’s ipsilateral hippocampus to less than 16 Gy while keeping the D100 under 9 Gy. These limitations are mostly due to the tumor’s anatomical position, form, and size, as well as the dose restrictions imposed by the closeness of nearby normal tissues. Moreover, it has been shown that unilateral hippocampal damage has a lower risk of significant cognitive impairment compared to bilateral hippocampal damage (36). Meanwhile, Goda et al. showed that an average dose of less than 30 Gy to one side of the hippocampus reduced the risk of long-term neurocognitive decline in patients (23). Therefore, we believe that limiting the average dose to the ipsilateral hippocampus to less than 30 Gy is a reasonable choice to strike a balance between protection and treatment. Simultaneously, the dosage to 100% of the contralateral hippocampus was not permitted to surpass 9 Gy, and the maximum hippocampal dosage was capped at 16 Gy.

The controversy and improvement of radiation technology have never stopped in the past decades. In a dosimetric study containing 20 patients with high-grade glioma, Canyilmaz E et al. reported a significantly greater reduction in hippocampus doses in IMRT when compared with VMAT (21). Recent research suggests that VMAT provides improved PTV coverage and better preservation of OARs, excluding the hippocampi, in glioma radiation than IMRT (37). In line with these findings, our study shows that VMAT can successfully minimize the irradiation dosage to the hippocampus, obtaining levels lower than those reported in prior studies.

There was a non-negligible limitation in this dosimetric feasibility study, which needs to be taken into account. This study evaluates the differential impact of two different radiotherapy plans on hippocampal radiation dose in patients with WHO grade II glioma. Nevertheless, damage to hippocampal function is caused by a combination of factors, not just the high radiotherapy dose to which one is exposed. Furthermore, the appropriate dose constraints that ensure hippocampal protection without compromising treatment effectiveness remain to be delineated. These pivotal considerations mandate further exploration through clinical research. In addition, the sample size of our patients was not sufficient to generalize our findings to the general patient population, despite the impressive advantages shown by our simulation of HS VMAT plans. The promising outcomes from our study are needed for further verification through clinical trials in order to determine the potential benefits of preserving cognitive functions in glioma patients undergoing HS VMAT.

## 5 Conclusion

The use of the HS VMAT plan is a feasible approach for the radiotherapy plan of WHO grade II glioma, and it can successfully reduce the dose delivered to the hippocampus without appreciably deteriorating the HI, CI, or the dosage delivered to the OARs. As such, it might, to some extent, help reduce the risk of cognitive impairment. The encouraging results of our study need to be further validated by clinical trials to confirm the benefits of HS VMAT plans in preserving cognitive functions in patients with glioma.

## Data Availability

The raw data supporting the conclusions of this article will be made available by the authors, without undue reservation.

## Supplementary material

Supplementary Figure 1 Optimization objectives of major structures for NHS-VMAT.

Supplementary Figure 2 Optimization objectives of major structures for HS-VMAT.
