# Supplementary figures and images for: Hippocampus-sparing volume-modulated arc therapy in patients with World Health Organization grade II glioma: a feasibility study
Source: Front Oncol. 2025 Jan 20;14:1445558. doi: 10.3389/fonc.2024.1445558 (PMC11788287; doi:10.3389/fonc.2024.1445558)

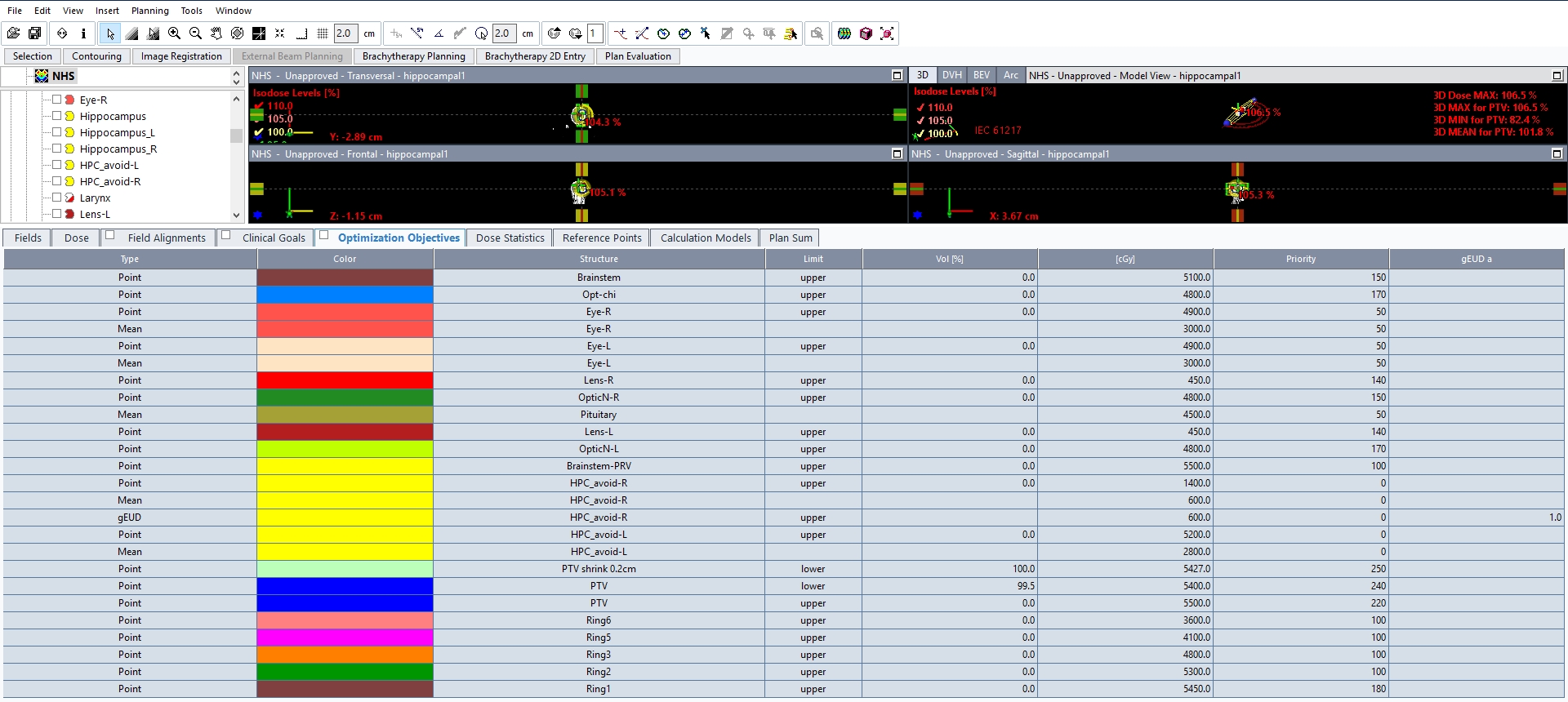

Supplement: Supplementary Figure 1 — Optimization objectives of major structures for NHS-VMAT. [file Image1.jpeg]

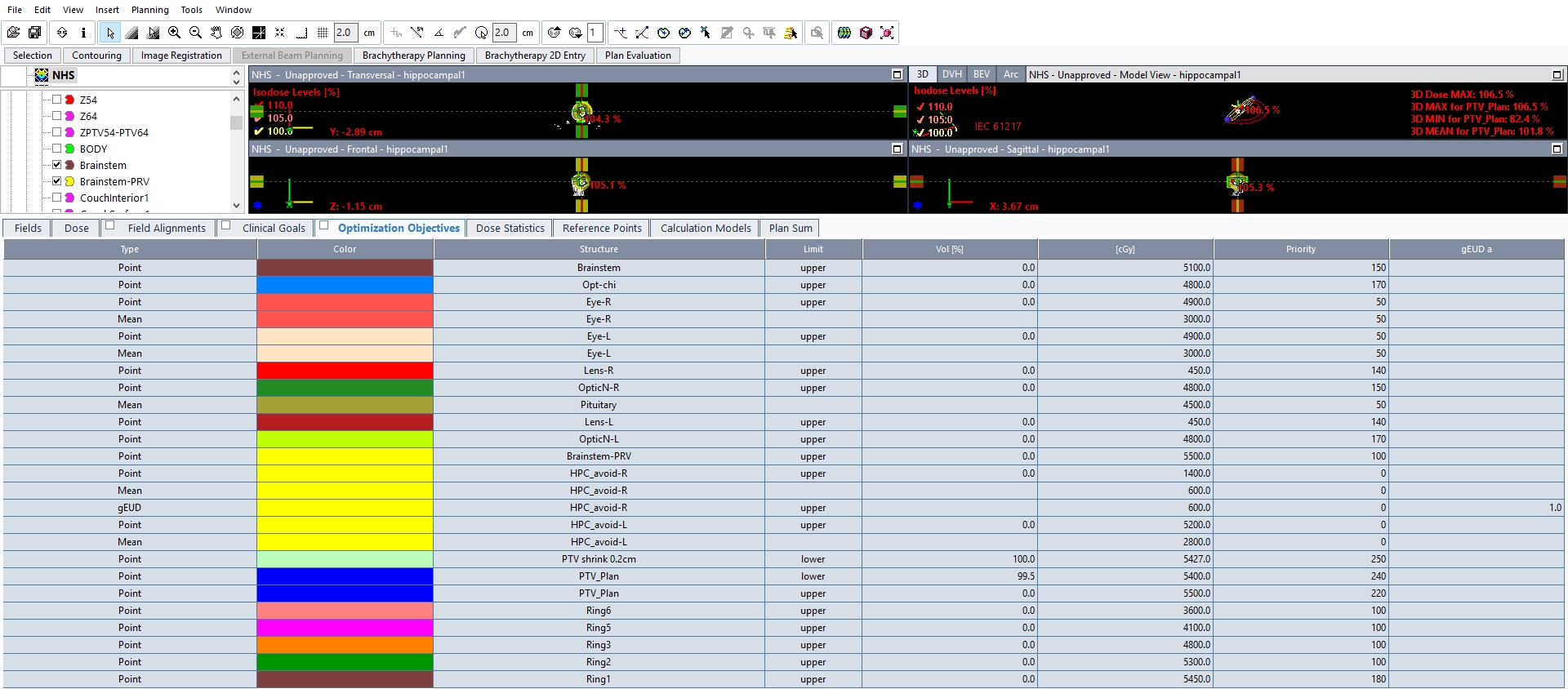

Supplement: Supplementary Figure 2 — Optimization objectives of major structures for HS-VMAT. [file Image2.jpeg]
